# EPIPDLF: a pretrained deep learning framework for predicting enhancer–promoter interactions

**DOI:** 10.1093/bioinformatics/btae716

**Published:** 2025-03-01

**Authors:** Zhichao Xiao, Yan Li, Yijie Ding, Liang Yu

**Affiliations:** School of Computer Science and Technology, Xidian University, Xi'an 710075, China; School of Management, Xi'an Polytechnic University, Xi'an 710075, China; Yangtze Delta Region Institute (Quzhou), University of Electronic Science and Technology of China, Quzhou 324000, China; School of Computer Science and Technology, Xidian University, Xi'an 710075, China

## Abstract

**Motivation:**

Enhancers and promoters, as regulatory DNA elements, play pivotal roles in gene expression, homeostasis, and disease development across various biological processes. With advancing research, it has been uncovered that distal enhancers may engage with nearby promoters to modulate the expression of target genes. This discovery holds significant implications for deepening our comprehension of various biological mechanisms. In recent years, numerous high-throughput wet-lab techniques have been created to detect possible interactions between enhancers and promoters. However, these experimental methods are often time-intensive and costly.

**Results:**

To tackle this issue, we have created an innovative deep learning approach, EPIPDLF, which utilizes advanced deep learning techniques to predict EPIs based solely on genomic sequences in an interpretable manner. Comparative evaluations across six benchmark datasets demonstrate that EPIPDLF consistently exhibits superior performance in EPI prediction. Additionally, by incorporating interpretable analysis mechanisms, our model enables the elucidation of learned features, aiding in the identification and biological analysis of important sequences.

**Availability and implementation:**

The source code and data are available at: https://github.com/xzc196/EPIPDLF.

## 1 Introduction

Enhancers and promoters are the two most important types of gene expression regulatory elements in mammals, especially humans. The efficient interaction between them ensures the accurate transcription of genes, thereby ensuring cell status and normal development. Erroneous associations between them can also lead to disease-related gene expression abnormalities. Therefore, exploring enhancer–promoter interactions is of great biological interest, but we know that genome-wide chromatin interaction mechanisms are complex (Ni *et al.* 2022). In particular, the emergence of high-throughput sequencing technologies such as Hi–C (Rao *et al.* 2014) and ChIA-PET (Heidari *et al.* 2014) has enabled us to more clearly understand the complex mode of action of EPI. In mammalian genomes, a gene’s promoter and its distal enhancer can be millions of base pairs apart, often not interacting with nearby enhancers. Instead, most enhancers skip adjacent genes to connect with distant promoters via long-range chromatin loops. The principles of chromatin interactions at the genome sequence level are unclear. Therefore, it is crucial to establish an effective computational method for identification and study EPI, and the large amount of data brought by high-throughput sequencing technology makes this feasible (Wei *et al.* 2021).

At present, many excellent calculation methods have been developed to identify EPI. Due to the huge amount of data, most calculation methods are based on deep learning technology (Liu *et al.* 2023, Qiao *et al.* 2024). In the early development of computational methods, researchers usually choose genomic features as input to the model, such as TargetFinder (Whalen *et al.* 2016), ChINN (Cao *et al.* 2021). TargetFinder proposed by Whalen *et al.* uses a large amount of genomic information, encompassing genomic peak data such as DNase-seq, DNA methylation, transcription factor ChIP-seq, histone modifications, CAGE, and gene expression data, to select and use on the classifier random forest (RF) (Cox, 1958) and support vector machine (SVM) (Boser *et al.* 1992, Wang *et al.*, 2023) were used for training data. Cao *et al.* proposed ChINN based on convolutional neural networks, which further achieved genome-wide prediction of chromatin interactions. In recent years of research, more and more work has chosen to use sequence data to train models. The main reasons are: 1. Compared with genome data, sequence data is out-of-the-box and does not require too many pre-processing steps. 2. The rapid development of the natural language field makes the process of training sequence data more complicated. technical means. Yang *et al.* (2017) developed PEP-Word, which uses word embeddings to extract features directly from sequences and trains a prediction algorithm for a boosted tree ensemble model. The results of their work demonstrate that genome-wide EPIs can be reliably predicted based on sequence features alone. In the same year, Mao *et al.* (2017) proposed EPIANN, which is a neural network architecture that utilizes the attention mechanism, and introduced positional feature encoding to further improve performance. In addition, more and more work is choosing to combine convolutional neural networks and recurrent neural networks on models. For example, the deep learning model SPEID (Zhuang *et al.* 2019) proposed by Singh *et al.* combines convolutional neural networks (CNN) and long short-term memory (LSTM) (Chen *et al.* 2022, Li *et al.* 2022). EPIVAN (Hong *et al.* 2020) proposed by Liu *et al.* also combines CNN and gated recurrent unit (GRU), and pretrains the model. Recently, numerous studies have used sequence data to explore deep learning for EPI prediction, achieving notable improvements in prediction performance. However, current deep learning predictors have not fully leveraged feature representation learning, particularly in identifying key sequence patterns crucial for understanding EPI mechanisms. Consequently, these models lack interpretability and fail to harness the impact of sequence-based approaches in EPI prediction (Yin *et al.* 2024).

In order to solve the above problems, we refer to some advanced technologies in natural language processing that are developing rapidly today, such as BERT (Devlin 2018, Ren *et al.* 2024, Zhang *et al.* 2024). Inspired by this, we treat DNA sequences as text data, and convert DNA sequences into ‘biological vocabulary’ by building a vocabulary. Therefore, we propose a model pretrained on large-scale genome sequences to learn biological context semantics, converting EPI ‘biological vocabulary’ into training data. In order to solve the lack of interpretability of deep learning, we try to apply both genomic data and sequence data to the model. We employ adversarial training and transfer learning to boost prediction performance and enhance model robustness. Benchmark results from seven cell line datasets show that our model substantially surpasses leading sequence-based approaches. Importantly, it offers interpretable predictions and analysis at the sequence level by examining local features using attention mechanisms. The model accurately and adaptively identifies sequence regions closely related to EPIs. Overall, our contributions can be summarized as follows:

1. We introduce a novel deep learning method named EPIPDLF, which is capable of training on pure sequence data and incorporates an additional gene data processing module.
2. We propose transfer learning and adversarial learning strategies to enhance model performance during testing and cross-cell line validation.
3. We utilize CNN modules and self-attention mechanisms to extract biologically meaningful motif sequences.

## 2 Materials and methods 

### 2.1 Dataset

In this study, we used the same EPI dataset as TargetFinder to assess our model and compare it to existing approaches. The dataset comprises EPIs from six human cell lines: GM12878 (lymphoblastoid cells), HUVEC (umbilical vein endothelial cells), HeLa-S3 (cervical carcinoma-derived cells), IMR90 (fetal lung fibroblasts), K562 (leukemia-derived mesodermal cells), and NHEK (epidermal keratinocytes). TargetFinder utilized annotations from ENCODE and Roadmap Epigenomics to identify active enhancers and promoters within each cell. For the analysis of these enhancers and promoters, high-resolution genome-wide measurements were performed for each cell line using Hi–C data. This enabled the classification of enhancer–promoter pairs into interacting (positive samples) and non-interacting (negative samples). For each positive sample, 20 negative samples were selected, resulting in a ratio of 1:20 between positive and negative samples within each cell line. Additionally, care was taken to ensure that the positive and negative samples exhibited similar distributions of enhancer–promoter distances. Table 1 provides detailed information on the datasets for each cell line. Moreover, we incorporated genomic information such as CTCF-binding sites, chromatin accessibility (DNase-I signals), and five histone marks (H3K27me3, H3K36me3, H3K4me1, H3K4me3, and H3K9me3). The processing of these genomic signals was conducted following the methods described by Chen *et al.* (2022).

**Table 1.** Sample distribution of all cell lines in the dataset.

Cell lines	EPIs	Non-EPIs
NHEK	1291	25 600
HUVEC	1524	30 400
GM12878	2113	42 200
IMR90	1254	25 000
HeLa	1740	34 800
K562	1977	39 500

### 2.2 Description of the proposed EPIPDLF

#### 2.2.1 Feature extraction

**Figure 1.**
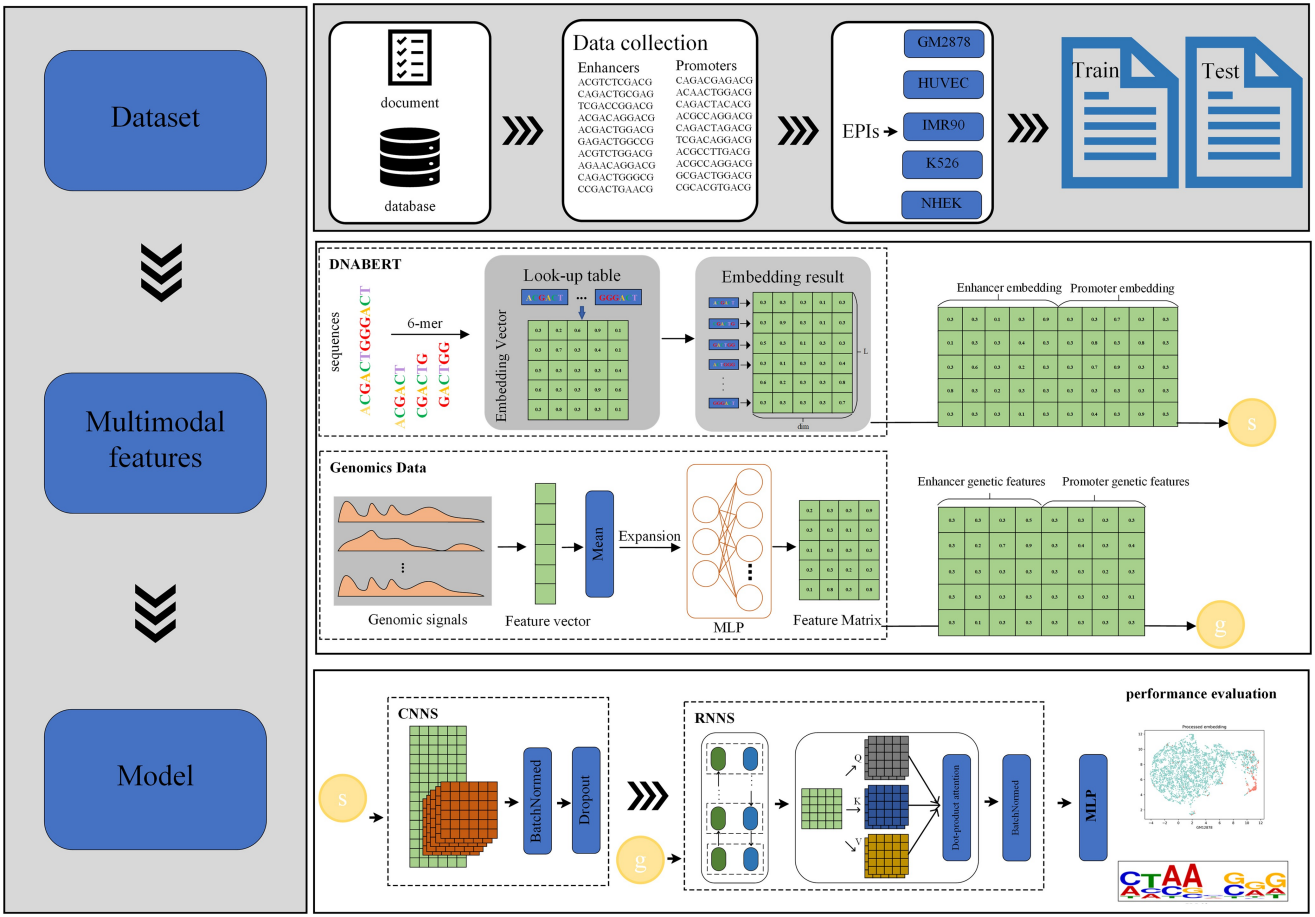
The workflow framework of EPIPDLF.

In this article, we use convolutional neural networks (CNNs) (Rakhlin 2016, Zulfiqar *et al.* 2024) and recurrent neural networks (RNNs) (Rakhlin 2016) to extract features, and to capture long-range dependencies among the features within the sequences, we further incorporate self-attention mechanisms, considering that the sequences are of considerable length (Li *et al.* 2021). Combining CNN and RNN is a common technique in deep learning for extracting features from sequential data. CNN excels at capturing meaningful local features and reducing the network’s parameter count efficiently. On the other hand, RNN utilizes its recurrent structure to model temporal relationships within sequential data. Next, we will outline the methods used for feature extraction. The complete framework of this research is depicted in Fig. 1.

*1D convolution layer*: the model uses a 1D CNN layer to generate embedding features from the sequence. Subsequently, MaxPooling is applied to perform down sampling and further reduce the dimensionality of the features.

*GRU layer*: Next, the features flow into a gated recurrent unit (GRU) (Chung *et al.* 2014), which is an improvement over conventional RNNs and has the ability to address the issue of long-term dependencies. The GRU is a form of recurrent neural network (RNN) intended for processing sequential data. It tackles the problem of long-term dependencies by incorporating gating mechanisms that regulate information flow. Here’s how GRU works.

Consider an input sequence (or time step) represented as *x* and a hidden state represented as *h*. GRU features two primary gates: comprising the update gate and the reset gate.

The reset gate (*r*) regulates the interaction between the previous hidden state (*h*) and the current input (*x*), influencing how much old information is discarded and how much new information is integrated. The formula for calculating the reset gate is provided as follows:

(1) $$r=\sigma (W_{r}\cdot [h_{t-1},x_{t}])$$

where $W_{r}$ represents the weight matrix associated with the reset gate, σ denotes the sigmoid function, and $\left[h_{t-1},x_{t}\right]$ represents the concatenation of the previous hidden state and the current input.

The update gate (*z*) regulates the inclusion of the previous hidden state (*h*) and the current input (*x*) in the current hidden state. It is computed as follows:

(2) $$z=\sigma (W_{z}\cdot [h_{t-1},x_{t}])$$

where $W_{z}$ is the weight matrix corresponding to the update gate. Next, we can compute the current candidate hidden state $\left(\tilde{h}\right)$:

(3) $$\tilde{h}=\tanh(W\cdot [r⊙h_{t-1},x_{t}])$$

where W is the weight matrix used to compute the candidate hidden state, and ⊙ denotes element-wise multiplication. Finally, we can compute the updated hidden state (*h*) using the update gate (*z*):

(4) $$h_{t}=(1-z)⊙h_{t-1}+z⊙\tilde{h}$$

where ⊙ denotes element-wise multiplication. This equation indicates that the current hidden state (*h*) is a weighted average of the previous hidden state (*h*) and the candidate hidden state $\left(\tilde{h}\right)$, with the update gate (*z*) regulating the weights between them. By utilizing both the update gate and the reset gate, GRU can determine which information to pass, ignore, or update, enabling it to handle long-term dependencies more effectively. This enables GRU to excel in a variety of sequence modeling applications, such as speech recognition and natural language processing.

*Attention layer*: multi-head self-attention mechanism is an attention mechanism used for sequence data modeling and is often used in natural language processing tasks. Below, I will detail the computational process of the multi-head self-attention mechanism using mathematical formulas. Suppose we have an input sequence, denoted as $X=[x_{1},x_{2},\ldots ,x_{n}]$, where $x_{i}$ represents the *i*th element in the sequence, with *n* denoting the length of the sequence. We need to calculate the correlation between each element and other elements in the sequence in order to obtain global contextual information. The multi-head self-attention mechanism captures different attention representations by introducing multiple attention heads. Suppose we have *h* attention heads, each head has its own parameter matrix for calculating attention weights. The calculation process of each head is divided into three steps: linear transformation, attention weight calculation, and weighted summation. First, we linearly transform the input sequence to map it to different query, key, and value spaces. For each attention head *i*, we define three sets of parameter matrices: $W_{i}^{n}\_q\in R^{d_{n}\times d_{\varphi }}$ is query transformation matrix, $W_{i}^{n}\_k\in R^{d_{n}\times d_{\varphi }}$ is key transformation matrix and $W_{i}^{n}\_v\in R^{d_{n}\times d_{\varphi }}$ is value transformation matrix. Among them, $d_{n}$ is the dimension of the input sequence, $d_{\varphi }$ is the dimension of the query key, and $d_{v}$ is the dimension of the value.

(5) $$q_{i}=W_{i}^{n}\_q\cdot x_{i}$$

 

(6) $$k_{i}=W_{i}^{n}\_k\cdot x_{i}$$

 

(7) $$v_{i}=W_{i}^{n}\_v\cdot x_{i}$$

Next, we calculate the attention weight of each element relative to other elements. We measure the correlation between two elements using a query-key dot product and normalize it through a scaling operation. Then, we perform a weighted sum of relevance and value to obtain the contextual representation of each element. For each attention head i, we compute the attention weight $A_{i}\in R^{n\times n}$:

(8) $$A_{i}=\text{softmax}\left(\frac{q_{i}\cdot k^{T}}{\sqrt{d\varphi }}\right)$$

Among them, softmax represents the normalization operation on the attention weight, and $\sqrt{d\varphi }$ is a scaling factor. Next, we perform a weighted sum of attention weights and values to obtain the contextual representation of each element. For each attention head *i*, we compute the context representation $c_{i}\in R^{n\times d_{v}}$:

(9) $$c_{i}=A_{i}\cdot v_{i}$$

Finally, we splice or average the context representation of each attention head to obtain the final multi-head self-attention representation C.

*Regularization mechanism*: To prevent overfitting during the training phase, we have employed a series of regularization techniques, primarily including dropout and batch normalization. Dropout is a regularization method employed to mitigate overfitting in neural network models. In each training batch, Dropout randomly sets the output values of some neurons to zero, that is, discards the contributions of these neurons. The purpose of this is to force the model not to depend on particular neurons, thereby enhancing the model’s robustness and capable of generalization. During the prediction phase, all neurons are retained and scaled by a retention probability to maintain model consistency. Batch normalization is a technique used in deep neural networks to normalize mini-batch inputs, setting each feature’s mean to near 0 and standard deviation to near 1. This accelerates training and enhances the network’s generalization. Specifically, for each mini-batch of input, Batch normalization first calculates the mean and standard deviation of the batch. Then, by performing linear transformation and translation operations on the input, the mean is adjusted to 0 and the standard deviation is adjusted to 1. Finally, a learnable scaling factor and translation factor are used to restore the original distribution of the data.

#### 2.2.2 Model training

##### 2.2.2.1 Loss functions and optimization method 

The proposed model EPIPDLF employs the binary cross-entropy (BCE) loss function, often utilized in binary classification scenarios. It quantifies the discrepancy between predicted values and actual outcomes, training the model by minimizing this difference as follows:

(10) $$\text{BCE}\text{Loss}=-\frac{1}{N}\sum_{i=1}^{N}\left[y_{i}\;\text{log}({\hat{y}}_{i})+(1-y_{i})\;\text{log}(1-{\hat{y}}_{i})\right]$$

Among them, $y_{i}$ is the real binary classification label, ${\hat{y}}_{i}$ represents the model’s predicted output, while N denotes the number of samples in a batch. The Adam optimizer (Diederik 2014) is employed to modify the learnable weights within the neural network. One of the benefits of using the Adam optimizer is its adaptability to different parameters.

##### 2.2.2.2 Pretraining strategy

To enhance the model’s generalization performance and its efficacy during cross-cell line validation, we employed a pretraining strategy. By integrating the training data from six cell lines, we constructed a substantial pretraining dataset, denoted as D. The model was pretrained on this dataset. Following the pretraining phase, the training process of the model can be described as follows:

1. Construct the pre-training dataset D, incorporating the training data from all cell lines.
2. Train model EPIPDLF on dataset D for 15 epochs, with a learning rate set to 0.001.
3. Fine-tune the pretrained model on the training data from each of the six cell lines, applying 10 epochs and maintaining a learning rate of 0.001 during this process.
4. Conduct predictions for the specific cell line and perform cross-cell line validation experiments.

##### 2.2.2.3 Adversarial learning strategy

Further, adversarial training is also incorporated into the model training process to enhance its robustness. The basic idea is to augment the training set with adversarial samples, enabling the model to learn from them during training. The introduction of adversarial learning necessitates the simultaneous fitting of adversarial samples during our standard training process, which may, to some extent, reduce the training speed. Adversarial examples are created using the projected gradient descent (PGD) approach. The PGD algorithm finds the most deceptive adversarial sample by iteratively applying gradient ascent and projection operations in the input space. The formula is described as follows:

(11) $$X^{*}=\mathrm{cli}p_{X,\in }\left(X+\alpha \cdot \mathrm{sign}\left(\nabla_{X}L\mathrm{oss}(X,Y)\right)\right)$$

Among them, X is the original input sample, $X^{*}$ is the generated adversarial sample, Loss(X,Y) represents the model’s loss function, while denotes the true label of the original sample. In every iteration of the PGD algorithm, the loss function Loss(X,Y) is computed to obtain the gradient with regard to the inputX. Then, the gradient is multiplied by the learning rate α and transformed into the direction of the gradient using the sign function sign(⋅). Next, the generated adversarial sample $X+\alpha \cdot \mathrm{sign}\left(\nabla_{X}L\mathrm{oss}(X,Y)\right)$ undergoes a projection operation to ensure that it stays within the range of ∈. The projection operation employs the function ${\mathrm{clip}}_{X,\in }(\cdot )$ to constrain the adversarial sample within the ϵ-range of the original sample X, thereby maintaining the acceptability of the adversarial sample.

### 2.3 Evaluation metrics

The dataset used for performance evaluation in this study is highly imbalanced. Therefore, we employ the area under the receiver operating characteristic curve (AUROC) (Hanley and McNeil, 1982, Liu *et al.* 2019, Zou *et al.* 2023, Li *et al.* 2024) and the area under the precision-recall curve (AUPR) (Davis and Goadrich 2006, Tang *et al.* 2021, Ai *et al.* 2023) as evaluation metrics. The receiver operating characteristic (ROC) curve illustrates the relationship between sensitivity (on the vertical axis) and the false-positive rate (1 - specificity, on the horizontal axis) across various thresholds. The area under this curve, AUROC, indicates model performance, with values closer to 1 (corresponding to the upper-left curve) reflecting better performance (Zhu *et al.* 2023). Since the ROC curve is unaffected by the distribution of positive and negative samples, AUROC is suitable for assessing models in imbalanced binary classification scenarios. Conversely, the precision–recall curve depicts the trade-off between precision (vertical axis) and recall (horizontal axis), emphasizing the balance between precision and recall for positive samples. The area under this curve, AUPR, measures model performance, with values nearing 1 (associated with the upper-right curve) indicating superior performance.

## 3 Results

### 3.1 The proposed EPIPDLF outperforms the state-of-the-art methods

To assess the performance of our proposed model EPIPDLF, we compared it against four leading predictors: PEP-WORD, SPEID, SIMCNN, and EPIANN. Each model was trained and tested using the same datasets for each cell line. The training process for each comparator predictor followed the methods described in their respective references. The AUROC and AUPR results for EPIPDLF and the four predictors across six cell lines are presented in Tables 2 and 3, respectively. EPIPDLF achieved the highest AUROC values in the HUVEC, HeLa, K562, and NHEK cell lines, with exceptions in GM12878 and IMR90. In Table 2, we observe that SIMCNN, apart from EPIPDLF, performs optimally in terms of the AUROC metric. Constructed using a straightforward CNN architecture, SIMCNN demonstrates that CNNs can effectively extract features associated with EPIs, thereby validating the appropriateness of utilizing CNN modules in our approach. Specifically, our model outperformed the second-best predictor by 0.2%, 1.5%, and 3.1% in HUVEC, HeLa, and NHEK, respectively. Similarly, EPIPDLF demonstrated superior AUPR performance across the six cell lines, excelling in HeLa and NHEK with improvements of 4.6% and 4.3% over the second-best model. Notably, PEP-WORD also exhibited high performance in AUPR. However, compared to all other models, PEP-WORD exhibits the poorest performance in terms of the AUROC metric, indicating that it places greater emphasis on predicting positive samples during evaluation. In summary, our model demonstrates superior performance compared to other predictors, achieving excellence in both AUROC and AUPR metrics.

**Table 2.** Comparison of AUC performance between EPIPDLF and other leading models on different cell line datasets.

Model/cell line	GM12878	HUVEC	HeLa	IMR90	K562	NHEK
EPIANN	0.919	0.918	0.924	0.945	**0.943**	0.959
SIMCNN	**0.941**	0.933	0.949	**0.951**	**0.943**	0.962
PEP-WORD	0.842	0.845	0.843	0.898	0.883	0.917
SPEID	0.916	0.904	0.923	0.915	0.922	0.950
EPIPDLF	0.939	**0.935**	**0.964**	0.936	**0.943**	**0.993**

Note: Bold fonts indicate optimal performance.

**Table 3.** Comparison of AUPR performance between EPIPDLF and other leading models on different cell line datasets.

Model/cell line	GM12878	HUVEC	HeLa	IMR90	K562	NHEK
EPIANN	0.723	0.616	0.702	0.770	0.673	0.861
SIMCNN	0.706	0.640	0.737	0.737	0.679	0.882
PEP-WORD	**0.807**	**0.760**	0.803	**0.868**	**0.836**	0.880
SPEID	0.773	0.523	0.797	0.732	0.771	0.852
EPIPDLF	0.788	0.730	**0.849**	0.779	0.755	**0.925**

Note: Bold fonts indicate optimal performance.

### 3.2 Contributions of pretrained strategy and adversarial training

**Figure 2.**
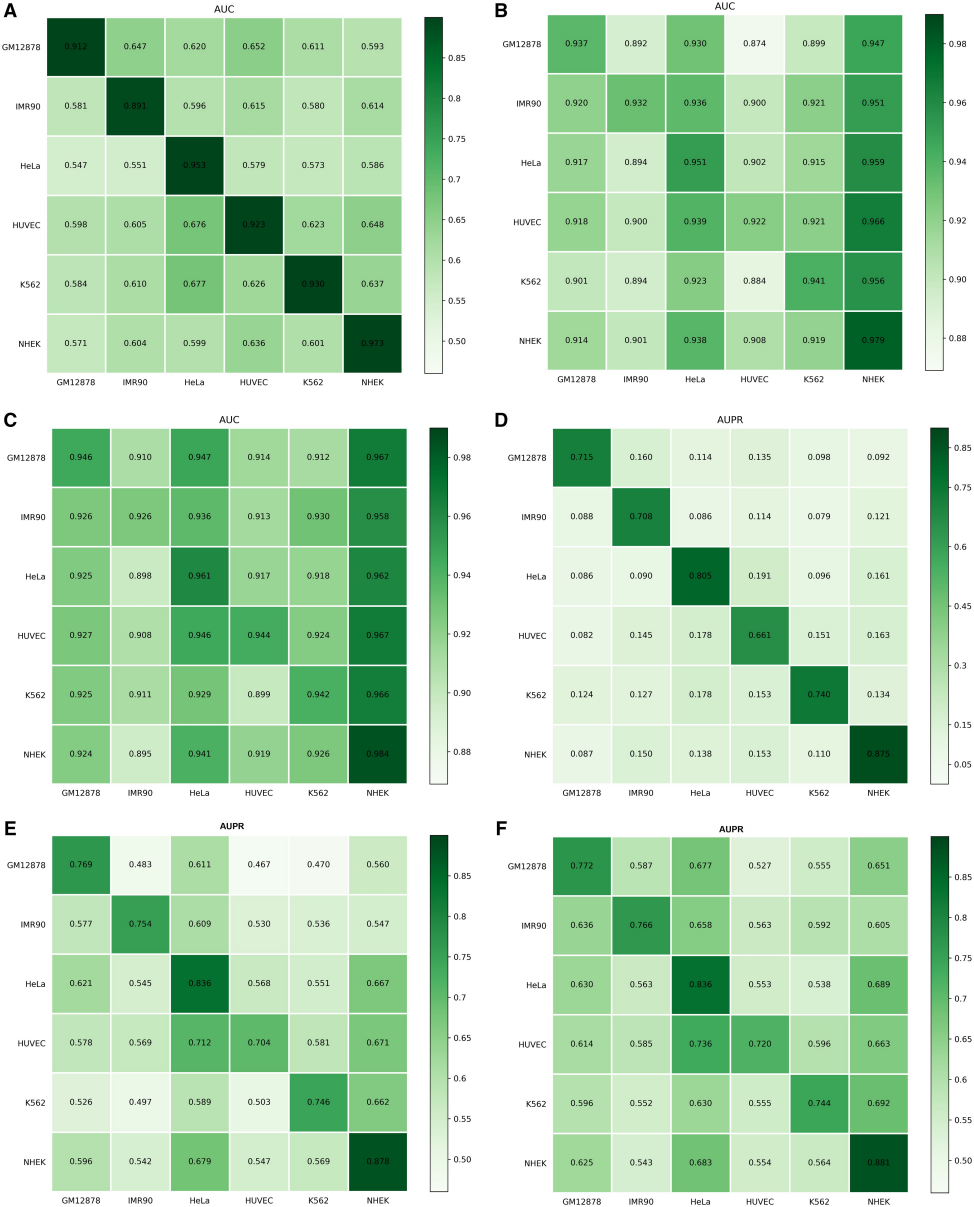
The impact of pretraining strategy and adversarial learning on cross-cell line model validation. (a) AUC value without any strategies. (b) AUC value after pretraining. (c) AUC value after pretraining and adversarial learning. (d) AUPR value without any strategies. (e) AUPR value after pretraining. (f) AUPR value after pretraining and adversarial learning.

Considering practical applications, EPI prediction models are often required to generalize across different cell lines. Therefore, the ability to predict EPIs across cell lines is particularly crucial. To improve the prediction capability across different cell lines, we suggest two strategies. The first strategy is pretraining, a common technique in transfer learning (Han *et al.* 2021) to improve model generalization. We initially pretrained our model on sequence data from all cell lines and subsequently fine-tuned the pretrained model on individual cell lines to derive the final models. In the previous section on model training, we provided a detailed description of the pre-training process. To further enhance the model’s cross-cell-line prediction capability, we integrated adversarial training (Madry 2017) into the pretraining process, which is a crucial component of our pretraining strategy. First, we aim to evaluate the effects of these two strategies on the validation of individual cell lines. Tables 4 and 5 illustrate the impact of the pretraining strategy and adversarial learning on a single cell line. The results indicate that the optimal performance is achieved when both strategies are employed simultaneously. Additionally, pretraining alone significantly enhances the AUC and AUPR values, demonstrating that both pretraining and adversarial learning are beneficial for model performance. To further validate the impact of these two strategies on cross-cell line validation, we conducted experiments, with results presented in the heatmap shown in Fig. 2. The performance improvement due to pre-training in cross-cell line validation is anticipated; notably, the enhancement from adversarial learning is more pronounced in cross-cell line validation than in the corresponding cell line validation. This is attributed to the substantial increase in robustness of the model following adversarial training.

**Table 4.** Demonstrating the impact of pretraining strategy and adversarial learning on AUC across six cell line datasets.

Strategy/cell line	GM12878	HUVEC	HeLa	IMR90	K562	NHEK
No strategy	0.912	0.923	0.953	0.891	0.930	0.973
Pre-training	0.937	0.922	0.951	**0.932**	0.941	0.979
Pre+adversarial	**0.946**	**0.944**	**0.961**	0.926	**0.942**	**0.984**

Note: Bold fonts indicate optimal performance.

**Table 5.** Demonstrating the impact of pretraining strategy and adversarial learning on AUPR across six cell line datasets.

Strategy/cell line	GM12878	HUVEC	HeLa	IMR90	K562	NHEK
No strategy	0.715	0.661	0.805	0.708	0.741	0.874
Pre-training	0.769	0.704	**0.836**	0.754	**0.746**	0.878
Pre+adversarial	**0.772**	**0.720**	**0.836**	**0.766**	0.744	**0.881**

Note: Bold fonts indicate optimal performance.

### 3.3 Genomic information effectively improves the performance of EPIPDLF

To validate whether genomic information serves as an effective feature input for EPIPDLF. Seven categories of genomic features were evaluated, including chromatin accessibility (DNase-I signals), CTCF-binding sites, and five histone modifications: H3K4me1, H3K9me3, H3K27me3, H3K4me3, and H3K36me3, following the methodology detailed by Chen *et al.* (2022). For the input genomic features, we initially used a multilayer perceptron (MLP) for reconstruction, followed by the application of multi-head attention to extract distal features. These were then fused with sequence features for the final prediction. As shown in Table 6, incorporating genomic features resulted in performance improvements across six different cell lines. This demonstrates that genomic information can indeed enhance the predictive performance of EPIs, provided that the genomic data is readily accessible.

**Table 6.** Improvement of model performance by gene data.

Cell line	Gene data	GM12878	HUVEC	HeLa	IMR90	K562	NHEK
AUC	Yes	0.939	0.935	0.964	0.936	0.943	0.993
	No	0.946	0.944	0.961	0.926	0.942	0.983
AUPR	Yes	0.788	0.730	0.849	0.779	0.755	0.925
	No	0.772	0.720	0.836	0.766	0.744	0.881

### 3.4 The biologically meaningful motifs learned by our CNN sequence learning module

**Figure 3.**
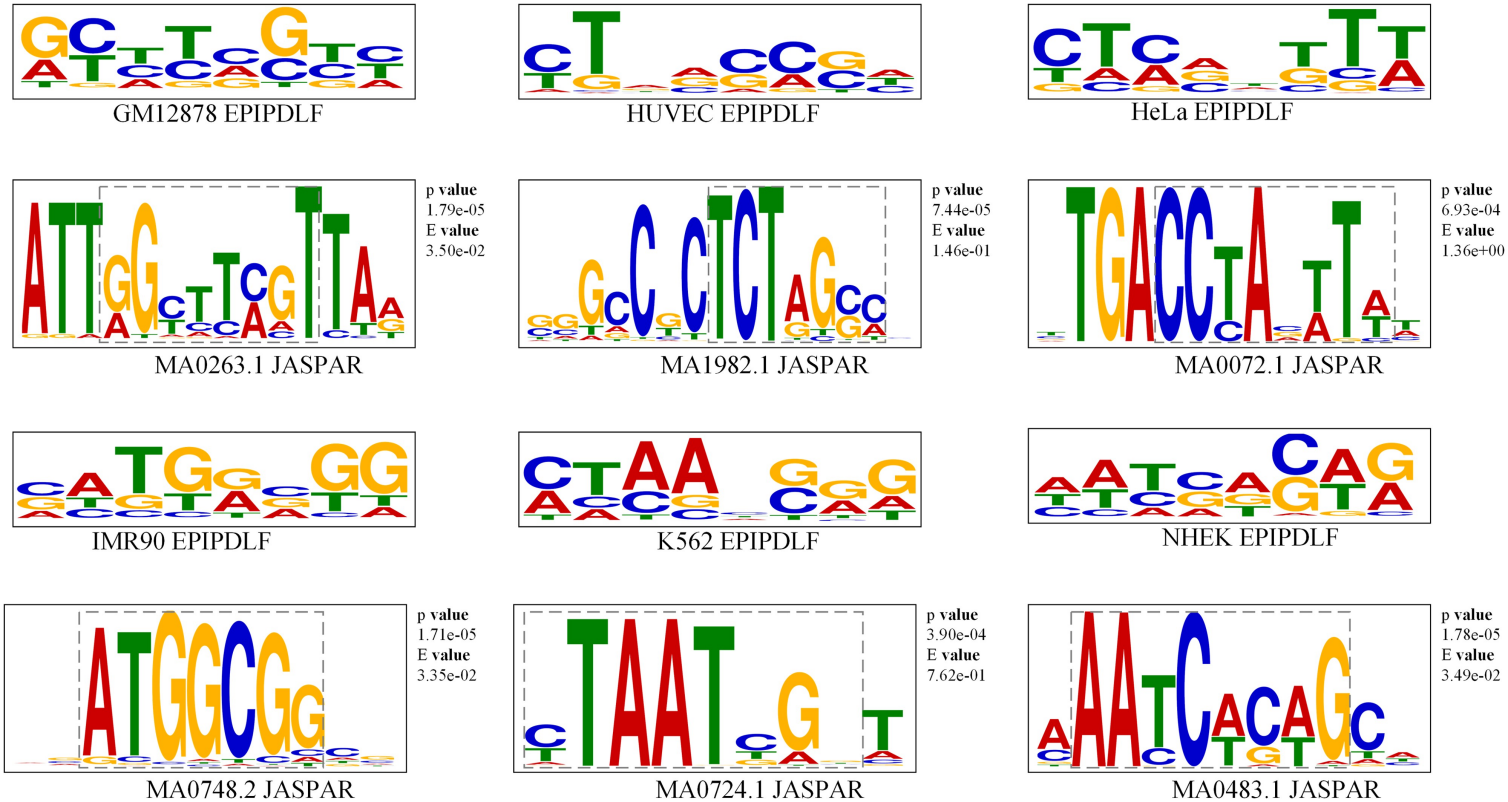
Comparison between motifs identified by the model in enhancer sequences and those in JASPAR.

**Figure 4.**
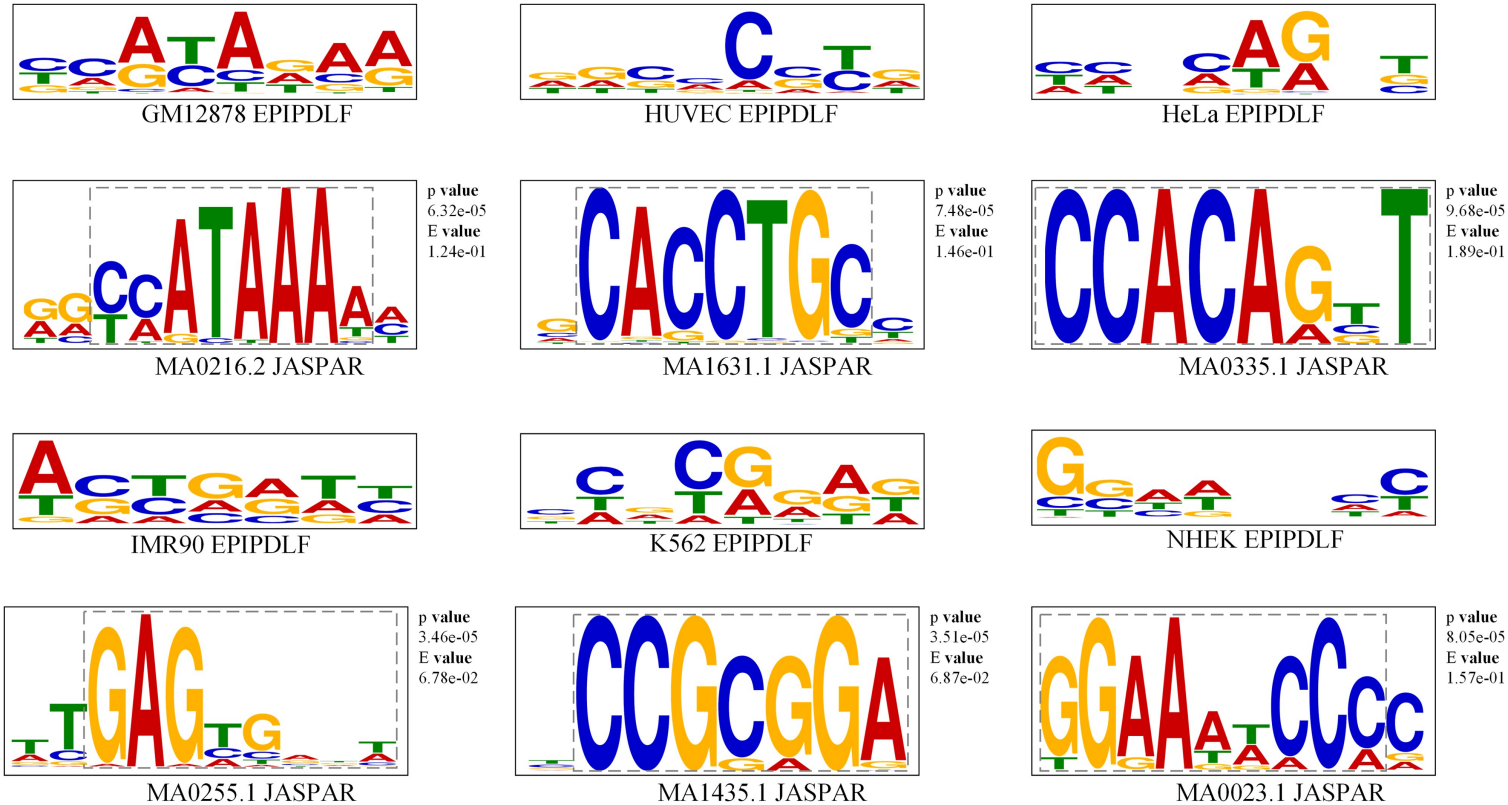
Comparison between motifs identified by the model in promoter sequences and those in JASPAR.

Next, we want to further explore whether the motifs learned by the model from sequence data have biological significance. Referring to reference (Jin *et al.* 2022), we extracted the motifs recognized by the model from the convolutional kernels corresponding to enhancers and promoters, and matched them with the motifs in the JASPAR (Sandelin *et al.* 2004) database. JASPAR is a well-known public transcription factor database. Furthermore, we used TOMTOM (Gupta *et al.* 2007) to calculate the similarity between the two motifs and use *P*-values to measure the similarity. The lower the *P*-value, the higher the consistency of the motifs. In Figs 3 and 4, the upper panel illustrates the motifs identified by EPIPDLF, while the lower panel displays similar motifs from the database. A comparison reveals that the patterns learned by the model from the sequences (enhancers and promoters) effectively match certain functional motifs in the transcription factor database, which have previously been reported to be associated with EPI. This result demonstrates that our model can effectively extract useful features from sequence data, and the high degree of matching with database motifs proves that the motifs identified by the model have sufficient biological significance.

## 4 Conclusion

In this article, we present EPIPDLF, an innovative method for identifying EPIs that relies solely on genomic sequence data. Compared to existing models, EPIPDLF incorporates pretrained DNABERT embedding matrices and a multi-head self-attention mechanism, enhancing its ability to capture latent features within sequences. Furthermore, we validated that related genomic information effectively aids EPI identification, suggesting that incorporating genomic data associated with EPIs into model training can be beneficial. In comparative experiments across six cell lines, EPIPDLF consistently outperformed other existing models, demonstrating its effectiveness. More importantly, we proposed two strategies, pretraining and adversarial learning, to enhance the model’s capability for cross-cell-line EPI prediction. Experimental results showed that pretraining significantly improves the model’s cross-cell-line generalization ability, while adversarial learning enhances model robustness, thereby improving its cross-cell-line prediction performance. Additionally, we validated whether the motifs learned by the convolutional neural networks (CNNs) layer of EPIPDLF possess biological significance. Our findings indicate that the model can accurately identify functional sequence features, and the newly identified motifs also have biological significance.

We recognize the potential of pretrained large models in bioinformatics. However, we did not fully exploit the pretrained DNABETRT; instead, we only extracted its embedding matrix. In future work, we aim to investigate whether more advanced pretrained models can be employed for biological sequences to obtain more informative pretrained DNA embedding matrices (Zou *et al.* 2019). Notably, DNABERT-2 (Zhou *et al.* 2023) has recently been published. EPIPDLF utilizes convolution and pooling to capture abstract local features without preserving the positional information of k-mers. Therefore, exploring how to incorporate additional features for EPI prediction remains a promising direction for future research.

Conflict of interest: None declared.

## Data Availability

The enhancer and promoter data in this article can be obtained at https://github.com/xzc196/EPIPDLF.
